# An Optimized Comparative Proteomic Approach as a Tool in Neurodegenerative Disease Research

**DOI:** 10.3390/cells11172653

**Published:** 2022-08-26

**Authors:** Rachel A. Kline, Lena Lößlein, Dominic Kurian, Judit Aguilar Martí, Samantha L. Eaton, Felipe A. Court, Thomas H. Gillingwater, Thomas M. Wishart

**Affiliations:** 1 The Roslin Institute, Royal (Dick) School of Veterinary Studies, The University of Edinburgh, Easter Bush Campus, Midlothian EH25 9RG, UK; 2 The Euan MacDonald Centre for Motor Neuron Disease Research, The University of Edinburgh, Edinburgh EH9 9AG, UK; 3 Section of Developmental Neurobiology, Department of Neurology, University Hospital Würzburg, 97080 Würzburg, Germany; 4 Center for Integrative Biology, Faculty of Sciences, Universidad Mayor, Santiago 8580745, Chile; 5 Geroscience Center for Brain Health and Metabolism (GERO), Santiago, Chile; 6 Buck Institute for Research on Aging, Novato, CA 94945, USA; 7 Edinburgh Medical School, Biomedical Sciences, The University of Edinburgh, Edinburgh EH8 9AG, UK

**Keywords:** proteomics, systems biology, experimental design, neurodegeneration, pathway analysis, data filtering

## Abstract

Recent advances in proteomic technologies now allow unparalleled assessment of the molecular composition of a wide range of sample types. However, the application of such technologies and techniques should not be undertaken lightly. Here, we describe why the design of a proteomics experiment itself is only the first step in yielding high-quality, translatable results. Indeed, the effectiveness and/or impact of the majority of contemporary proteomics screens are hindered not by commonly considered technical limitations such as low proteome coverage but rather by insufficient analyses. Proteomic experimentation requires a careful methodological selection to account for variables from sample collection, through to database searches for peptide identification to standardised post-mass spectrometry options directed analysis workflow, which should be adjusted for each study, from determining when and how to filter proteomic data to choosing holistic versus trend-wise analyses for biologically relevant patterns. Finally, we highlight and discuss the difficulties inherent in the modelling and study of the majority of progressive neurodegenerative conditions. We provide evidence (in the context of neurodegenerative research) for the benefit of undertaking a comparative approach through the application of the above considerations in the alignment of publicly available pre-existing data sets to identify potential novel regulators of neuronal stability.

## 1. Introduction

Degenerative diseases of the central and peripheral nervous system are universally a significant public health priority, accounting for nearly 10% of the global health burden. These conditions vary broadly in their regional pathology, age of onset, and underlying aetiology, and the majority are currently without available or limited therapeutic options. While in recent years, there have been few successes in the translation of research to therapies, notably for monogenetic conditions such as gene-targeted treatments for Spinal Muscular Atrophy (SMA) and the CLN2 form of Batten disease [1,2,3,4,5,6,7] and oral supplementation therapy for hereditary sensory and autonomic neuropathy disease (HSAN1) [8], it remains even more difficult to design targeted therapies for the majority of idiopathic non-heritable neurodegenerative conditions. These aetiologically complex diseases, including most forms of amyotrophic lateral sclerosis (ALS), Alzheimer’s disease (AD), Parkinson’s disease (PD), and other dementias, therefore remain invariably life-diminishing.

Historically, the most promising success stories in the characterisation and treatment of these comparably clear-cut monogenetic conditions such as SMA and CLN2 disease have only prevailed following decades of challenges building on an initial characterisation of the causative gene or mutation. Indeed, within both the SMA and the lysosomal storage disorder fields (of which CLN2 is amongst a family of over 13 heritable diseases), the relevance of even well-established animal or in vitro modelling strategies remains contentious, rendering it difficult to define disease-specific pathophysiology at even a gross neuroanatomical level, much less on a cellular or molecular basis [9,10,11,12]. In addition, the translational relevance of findings derived from such models remains difficult to ascertain [13]. This is further complicated by the availability of samples; as these monogenetic neurodegenerative conditions remain inherently rare, tissue samples for validation studies are consequentially difficult to obtain. Finally, other challenges may lie in the specific biochemical configurations of the affected gene and subsequent protein product to hinder the development and delivery of gene-specific therapies; a prominent example exists in the challenges surrounding the functionally elusive transmembrane protein CLN3, inherited mutations that cause another form of neuronal ceroid lipofuscinosis [14].

For non-heritable conditions, the impediments in defining relevant therapeutic targets remain even more elusive. The characteristic symptomology of Alzheimer’s (AD) and Parkinson’s (PD) diseases was originally described over a century ago [15,16]. While in recent years, significant advances in techniques to define the histopathology of these conditions has enabled milestone discoveries in understanding the cellular consequences of AD, PD, and other dementias down to the synaptic level [17], elucidating the molecular basis underpinning these degenerative processes remains highly complicated. For example, while recent advances in GWAS studies have uncovered a number of epidemiological risk factors, including the critical discovery of the ApoE4 allele, the means by which these risk factors contribute to the likelihood of developing synaptic loss accompanying cognitive decline remains poorly understood [18,19,20,21]. Consequentially, the translational relevance of murine models, in particular to human disease, is also frequently challenged [22,23]. While the global prevalence of conditions such as AD and PD generates a relative availability of post mortem specimens, the heterogeneity of the human population, even between age-matched, sex-matched individuals within a contained geographical population, ensures difficulty in delineating any disease-causing molecular cascades [24,25]. It is, therefore, perhaps unsurprising that the majority of these “spontaneous” conditions remain without a known modifying treatment or cure.

For these progressive neurodegenerative conditions in which both a well-defined cause and treatment options remain elusive, the application of -omics techniques to untangle the causative molecular pathophysiology harbours enormous potential. Through -omics-based experimentation (and systems-based analyses, to be discussed subsequently), it is possible to not only pinpoint alterations in the expression levels of specific molecules (i.e., genes, transcripts, proteins, etc.) but also to quantify broader dynamics in the genome, transcriptome, or proteome (or indeed, metabolome, lipidome, etc.). Discovery-based experiments—in particular, proteomic screens, which through the identification and relative or absolute quantitation of proteins, generate arguably the most accurate overview of “functional” molecular dynamics—may represent the most straightforward approach to uncovering disease-specific molecular pathology. For example, profiling in vitro or in vivo models or post mortem patient samples may be useful in determining any therapeutically targetable elements within these pathological cascades [26,27].

Nevertheless, despite recent advances in proteomic technologies, their translational application to the neurodegenerative disease field—particularly toward the aforementioned conditions with ambiguous or complex aetiologies—remains in its infancy. In the following review, we outline a number of considerations, caveats, and suggestions for designing and analysing proteomic experiments from in vitro or in vivo models, or else post mortem tissue from progressive neurodegenerative conditions, in order to extract maximum biological relevance and, therefore, therapeutic potential.

## 2. Proteomics as Applied to Neurodegenerative Disease Research

“-Omics” screening technologies, including proteomics, enable the generation of a comprehensive molecular “fingerprint” of a specific tissue or cell population through health and disease or following experimental manipulation. Assembling this “fingerprint,” numbering in the several thousands of protein identifications using modern mass spectrometry methods, to tens of thousands of transcripts identified through RNAseq or microarray techniques, and before tracing it through, for example, the time course of disease in a model organism results in enormous expression datasets ripe for contextualisation. The integration and analysis of these large -omics datasets have subsequently given rise to the systems biology field, in which predictive algorithms attempt to determine how any molecular alterations identified may interact to promote phenotypic alterations reflected in vitro or in vivo.

Recent major advances in sample preparation and mass spectrometry techniques have revolutionised the comprehensiveness of proteome coverage, particularly within whole-tissue experiments. Indeed, to date, a PubMed search of the terms “proteomics AND neurodegeneration” yields 1459 publications, while searches for “proteomics AND Alzheimer’s” and “proteomics AND Parkinson’s” yield 2871 and 1447 results, respectively. Despite this, there has been a lack of accompanying advancement in the identification of translatable targets within the fields of neurodegenerative diseases. Unfortunately, studies aiming to generate proteomic profiles of in vivo or in post mortem tissues are subject to the inevitable challenges plaguing most neurodegenerative disease fields. These include but are not limited to the unreliability of animal models, scarce tissue availability, and inappropriate sample handling or storage. On the other hand, while in vitro studies benefit from the ability to devise more tightly controlled and genetically faithful recapitulations of disease-causing or disease-associated alleles, they generally lack the capacity to emulate multicellular involvement; this is a particular drawback in neurodegenerative contexts, as the cross-talk between neurons and astrocytes, glia, or oligodendrocytes appears to be a critical driver of disease [28,29,30,31,32,33].

Additionally, proteomics studies themselves (or indeed, any -omics type experimentation) introduce additional challenges. Despite technological advances yielding a greater coverage of the proteome than ever before, the ability to process these larger datasets in order to conduct meaningful analysis and subsequent validation experiments often proves a significant hurdle. Finally, perhaps the most crucial difficulty lies in studying translatability; publishing a screen that reports tens, hundreds (or indeed thousands) of protein alterations ultimately does not inform upon how these protein(s) may be contributing to disease-specific neurodegeneration. For example, of the 2871 publications to date featuring the keywords “proteomics AND Alzheimer’s”, only 843 (<30%) include data demonstrating subsequent applicability in vivo beyond simple validation experiments (e.g., immunoblotting) to verify screen results. It is, therefore, highly evident that incorporating quantitative proteomics into a meaningful study with translatable results requires careful experimental design, numerous considerations in generating and handling proteomic data, and relevant analyses at a systems level.

It is also important to note that the majority of -omics centred publications, even those featuring a translation-forward conclusion, tend to be established toward a specific gene/transcript/protein candidate, often with a bias toward known “familiar favourites” in the established literature. While this “cherry-picking” approach may provide important internal validation for the study design, the researcher risks not only biasing their study in favour of these candidate(s) but also ignoring the remainder data, which, upon extrapolation to a network level, may provide greater insight into the biological events occurring within their study.

Consequentially, we believe that this single-track approach (with a field-by-field internal focus on self-reinforcing targets of existing interest) may contribute, in part, to the statistic of over 90% of clinical neuroscience trials failing before Phase II [10,34]. More optimistically, we instead suggest that with (1) considered experimental design, (2) unbiased analytic approaches, and, optionally, (3) comparative alignment with other relevant -omics datasets, the potential to overcome this translational gap and yield greater insight into singular or conserved pathways of disease-specific degeneration is possible. In this manuscript, we seek to reinforce the importance of careful experimental design in proteomics studies, from sample collection to database searches for peptide identification, in order to yield high-quality, translatable results.

Additionally, we suggest that the majority of contemporary proteomics screens are hindered not by technical limitations such as low proteome coverage but rather by insufficient analyses. We, therefore, emphasise the variables within a standard analysis workflow that should be deliberated and adjusted for each individual study. These include considerations into when and how to filter proteomic data to provide options for analyses of dataset(s) either in their entirety or in trend-wise analyses for biologically relevant patterns. Finally, we recognise the difficulties imposed in the modelling and study of neurodegenerative conditions and therefore provide evidence for the benefit of taking a comparative approach through a considered alignment of published or collaborative data sets to look for common regulators of neuronal stability. As proof of principle, we conclude by offering an example “walk-through” of data identifying conserved regulators of neuronal stability derived from the comparison of two distinct, previously published proteomic datasets.

## 3. Considerations, Not Limitations

**Figure 1:**
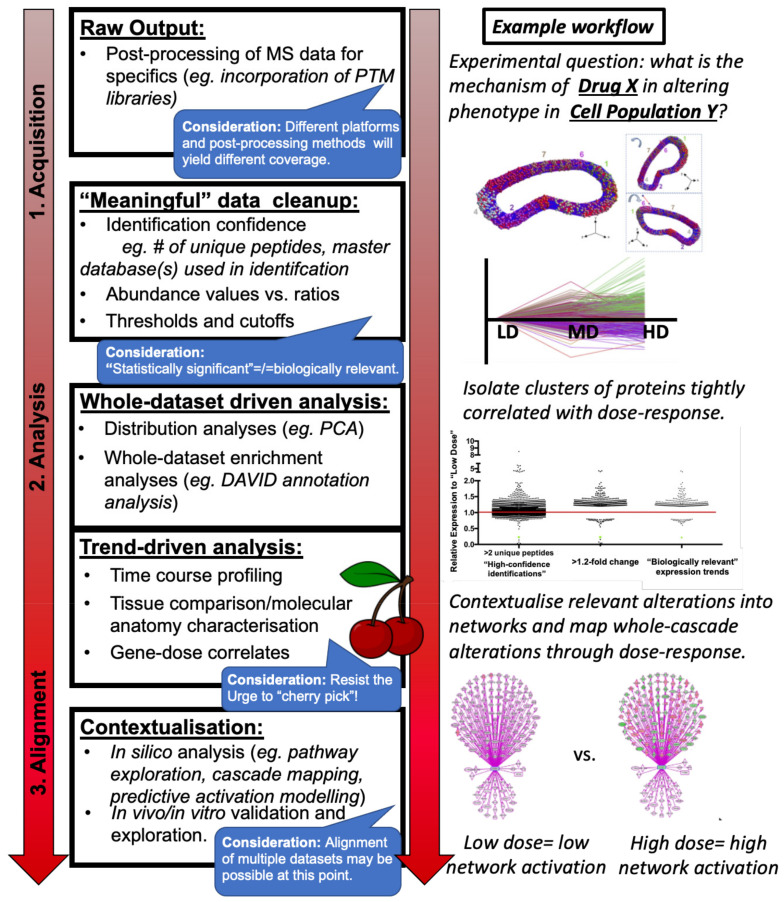
Schematic summarising workflows described to optimise -omics style experimental design and data analysis.

Broadly speaking, the experimental strategies for mass spectrometry-based proteomics, particularly the bottom-up proteomics studies largely referenced in this manuscript, may be summarised by the following workflow:

1. Sample collection and preparation of in vitro cells, in vivo tissue from animal models of disease, or post mortem tissue from end-stage patients or age-matched controls.
2. Protein identification and absolute or relative quantitation within sample(s) including appropriate statistical analysis and multi-database searches, generally conducted within one of several commonly used proteomics software (Figure 1: Raw Output).
3. Post-mass spectrometry filtering, if appropriate (Figure 1: “Meaningful Data Cleanup”) and:
4. Downstream data analysis methods of the whole dataset (Figure 1: Whole dataset-driven analysis) or isolated subsets (Figure 1: Trend-driven analysis) in order to posit biological meaning from the protein abundance data generated by the mass spectrometry experiment(s) (Figure 1: Contextualisation).

Each element of this workflow includes study-specific caveats of particular interest to neurodegenerative researchers that should be considered and incorporated on an individual basis for each experiment. Below, we briefly summarise each element of this workflow and include points of interest for neurodegenerative disease research based on studies generated both within our own laboratories and in the published literature.

### 3.1. Sample Collection

In designing a proteomics-based study, it is important to consider the source material to be profiled, particularly given the caveats in neurodegenerative disease research described previously. As always, an appropriate control must be employed, including, if possible, an isogenic control cell line [35]; however, it may also be useful in proteomics-based studies to design a “scalable” experiment, particularly in the aim of profiling models of neurodegenerative disease. For example, while studying in vitro models, juxtaposing control cells with those harbouring an exogenously expressed construct recapitulating a known mutation at low, medium, and high levels could be utilised to provide a means of extracting proteome alterations directly consequential to the mutation of interest. As with all genetic manipulation strategies, off-target effects or a potentially confounding molecular response due to the expression of a construct past physiological levels must be considered and accounted for. However, we also suggest that the stratification method described above, through which a core “dose–response” profile may be generated through profiling differential levels of exogenous expression and isolating any trends correlative with construct levels (see Figure 1: Trend-driven analysis), may greatly reduce this potential compared to a simple “A versus B” comparison. A parallel example in profiling a cellular response to drug treatment by dosage scaling is illustrated in Figure 1 above. Similarly, in using rodent models of heritable disease, it may be prudent to include heterozygous animals in the study in order to study the “dose–response” result of, for example (theoretically), 100% expression of the full-length protein (wild-type), 50% of full-length, 50% truncated (heterozygote), 0% full-length, and 100% truncated (homozygote). For a similar example of how designing an experiment may enable stratification by dosage levels in order to profile a cellular response to drug treatment, please refer to Figure 1.

For time-course studies utilising animal models or in profiling post mortem tissue, one should take into account that end-stage neurodegenerative disease may feature severe or chronic processes resulting in cell death and synaptic loss [27,36]. It is, therefore, possible that any alterations detected at this point are either the universal “death signature” of a dying neuron (and therefore not representative of disease-specific pathogenesis) or else, sampling only the “survivors” and thereby detecting correlates of neuronal resistance to degeneration. At this point, it would be prudent to include comparisons with earlier stages of disease progression, if possible, or to cross-check with other studies with collaborative or publicly available similar datasets (see the comparative section below). These comparisons would better establish if the alterations identified are disease-specific vs. global indicators of neurodegeneration, or moreover, if they are part of pro-degenerative cascades vs. potential mediators of neuronal survival.

Particular care should be taken in dissecting the neural tissue to be utilised in the study in order to minimise the potential for any expression profile “alterations” to be merely the consequences of differences in molecular anatomy. A study by Eaton et al. revealed that different segments of the same murine sciatic nerve harboured markedly different expression levels in proteins commonly used as “housekeeping” proteins in western blot studies, such as GAPDH and beta-actin [37]. This same consideration should be taken while isolating specific brain regions or spinal cord regions; for example, while laser-capture microdissection is often a prohibitively expensive procedure for most proteomic studies, careful microdissection of structures as small as the murine neuromuscular junction is feasible, as are fractionation protocols for tagged cell populations [9,38]. In situ techniques currently gaining traction, such as MALDI-imaging mass spectrometry, which utilises a UV laser for the desorption and ionisation of peptides or other analytes from a tissue section, offer the potential for highly specific proteomic analysis of microregions, but this is not discussed within the scope of this review.

In terms of extraction methods themselves, a number of studies have demonstrated enormous variability in proteome coverage merely based on the method of extraction, such as the use of a Dounce or motorised hand-held homogenizer versus a total tissue dissociation tool [39,40]. Different dissociation techniques and implementation of ice-cold temperatures must be particularly considered in preparations for non-neuronal cell types such as microglia, which due to high reactivity in the event of an “insult” such as enzymatic digestion and physical disruption, may exhibit a potentially confounding proteomic profile upon analysis [41]. Similarly, storage of both pre-extracted tissue and extracted protein may potentially affect study quality, with storage at −20 °C resulting in marked degradation of protein compared to −80 °C [17,42].

Choice of lysis buffer may also impact comprehensiveness of coverage, particularly in high-lipid tissues such as neuronal tissue. To obtain a wider class of both hydrophilic and hydrophobic proteins effectively extracted typically, lysis buffers with strong denaturants or detergents may be employed. While ionic detergents such as sodium dodecyl sulphate (anionic organosulphates) or sodium deoxycholate (ionic detergent) or chaotropes/chatropic agents (which act to disrupt hydrogen bonds) such as urea offer effective solubilisation of wide range of proteins including recalcitrant membrane proteins, typically these are buffer-exchanged or diluted prior to proteolytic digestions and downstream chromatographic separations. Additionally, protein precipitations methods using either TCA or acetone are effective strategies for delipidation of lipid-enriched tissues and selective removal of other undesirable small molecules from the protein samples. Depending on the sample handling procedures or on the experimental objectives (e.g., phosphoproteomics), either protease or phosphatase inhibitors are required to maintain protein quality throughout the procedure.

Proteomic studies designed toward identifying biomarkers from peripherally accessible samples such as blood, CSF, or urine may require additional processing steps in order to maximise proteome coverage (such as albumin depletion for blood or plasma samples); however, this is not discussed in this manuscript [43,44].

### 3.2. Mass Spectrometry Experiment and Proteomic Data Analysis

In the bottom-up proteomic workflows discussed in this manuscript, the purpose of the mass spectrometry experiment is to generate protein identifications with accompanying quantitative values (i.e., identification and abundance values). The analyses here refer to the spectral identifications, data pre-processing, and normalisation analysis required for confidence in generating these IDs and abundance values. They also include the statistical methods and filtering processes commonly used prior to more in-depth downstream analyses (described in a separate section).

#### 3.2.1. Quantitative Proteomic Profiling via Mass Spectrometry

LC-MS-based bottom-up proteomics involves data generation by either data-dependent acquisition (DDA), where individual peptide precursor ions are selected for fragmentation or by data-independent acquisition (DIA) where all peptide precursors within a specified mass window (typically 10–20 m/z) are concurrently fragmented.

The first step in the identification workflow involves spectra-peptide matching in order to establish a peptide ID. Multiple algorithmic techniques are used for deriving peptide IDs (and subsequently proteins IDs) and can be broadly classified as either database search-based or de novo sequencing-based.

Over the course of last two decades, several database searching algorithms have been published utilising probability-based scoring or hidden Markov scoring function for spectral search, peptide and protein scoring, and protein inference. A comprehensive listing of these algorithms is beyond the scope of this review, but some of the major ones that are widely used by the proteomics community are MASCOT [45], OMSSA [46], MyriMatch [47], Andromeda [48], Comet [49], MSFragger [50], etc. These algorithms are available either as a commercial license or as integrated into open-source proteomics data analysis pipelines. Most of these algorithms provide a measure of false-discovery rate (FDR) at peptide or protein levels frequently by doing a target-decoy search strategy [51].

Appropriate selection of search criteria, such as precursor and fragment mass tolerance, fixed or variable modifications, and allowance of missed cleavage, are both instrument and sample dependant and, therefore, must be optimised for individual experiments. Sample treatments, proteases used, and stable isotope labelling strategies can bring about chemical and structural changes to one or more amino acids on a peptide sequence, and therefore the m/z of peptides and these should be taken into consideration during database searches. One of the pre-requisites for database searching of the fragment ion spectra is the selection of an appropriate sequence database as search engines can report a match only if the sequence is present in the database. Generic protein databases such as UniPROT or Ensembl genome browser offer tools to selectively download species-specific protein/DNA sequences that may be used with search engines.

While the most commonly used computational method for peptide identification remains the database search, direct sequencing of fragment ion spectra to derive peptide spectral match is also used as a database-independent alternative. The enhanced sensitivity and resolution of a modern mass spectrometer and the resulting higher quality of spectra now enable direct de novo sequencing of a large number of spectra [52]. De novo sequencing has the added advantages of identifying mutated protein sequences, amino acid substitutions, and unexpected post-translational modifications, and emerging methods based on deep learning neural network models [53] offer improved sequencing accuracy.

Mass spectrometry has evolved as the method of choice for unbiased quantification of a large number of proteins/peptides from biological samples, and several MS-based workflows have been developed over the last two decades. Considerable development in instrument resolution, sensitivity, and software solutions now enable both relative and absolute quantifications of proteins. These are performed by either using stable isotopic labelling methods or ‘label-free’ approaches. Stable isotopic labelling allows multiple but differently labelled samples to be combined and analysed as a single sample, thereby minimising run-to-run variability to a great extent as compared to label-free methods, which require individual samples to be run separately through LC-MS. The labelling methods can be broadly classified as metabolic (SILAC, N15, etc.) or chemical (dimethyl, iTRAQ, or TMT, to name a few) depending on how the labels are incorporated onto the protein or peptides. While MS1-based quantification workflows (label free, SILAC, dimethyl labelling, etc.) measure intensities of peptide precursors, MS2-based methods (iTRAQ, TMT, etc.) rely on the diagnostic fragment ions. Some of the MS2-based labelling strategies (e.g., TMT) allow higher order multiplexing allowing up to 16 samples in a single run.

#### 3.2.2. Pre-Processing Analysis and Normalisation

In the quantitation processes described above, the final abundance of a protein is derived from peptide information. Regardless of the quantification methods used, it is essential to adjust the cut-off for the minimum peptide numbers required to generate this composite protein abundance value (see Figure 1: Acquisition). Typically, most bottom-up quantitative proteomics workflows also employ a number of normalisation processes at this stage prior to further analyses in order to correct for any stochastic inter- and intra- run variability. This may be attributed to the general operation of the instrumentations or retention time drift during chromatography or batch-effect at multiple times on different days on large cohort experimental runs. Appropriate normalisation maximises the potential for reported protein expression alterations and interpretation through downstream analyses to be biological events capable of validation in vivo or in vitro, as well as enhancing the capacity for the researcher’s visualisation and contextualisation. For example, the enormous variation in endogenous expression levels within the “wild-type” proteome, with the presence several “superabundant proteins” (e.g., albumin or myosins), often skew the majority remainder of other protein abundances close to “zero.” For this reason, logarithmic transformations are often applied to raw abundance values prior to further normalisation steps. These normalisation methods are based on various statistical assumptions and include linear regression-based normalisation, variance stabilisation normalisation, and median normalisation [54,55,56] and must be tailored individually for each experiment depending on the labelling process employed and tissue profiled [38,44,54,57,58].

#### 3.2.3. Post-Processing Analysis

Within the quantitative proteomics (and indeed, general -omics) fields, common workflows often conclude with statistical analyses such as t-tests or analysis of variance tests (ANOVA) (with another established FDR threshold) between experimental groups performed on the normalised data. However, the appropriateness of these traditional methods is increasingly contested, with Bayesian methods gaining increasing recognition for circumvention of proteomics-specific issues that traditional t-test methods generate, such as disproportionately large *p*-values due to fewer *n* numbers of experimental groups. Although the multiplexing capacity of methods such as TMT is increasing markedly, the abundance of proteomics datasets is still far fewer than those derived from microarray or RNAseq [59].

#### 3.2.4. Data Deposition

Perhaps as a consequence of the relative modernity of MS-based proteomics compared to more “mature” -omics techniques such as genomics, the practice of data deposition remains frustratingly nonstandardised and, for some journals, entirely nonmandated [60]. Indeed, this lack of open accessibility in proteomics data (and indeed, to a lesser extent, other -omics) remains a major impediment to scientific transparency.

In addition to these obvious hindrances toward research progress, we also suggest that this lack of data sharing practice in neuronal proteomics stifles otherwise invaluable opportunities to perform, for example, re-analyses or else comparative analyses customised to a novel research question (as described below in Section 3.3) [61,62].

A number of proteomics repositories, reviewed thoroughly in Vaudel et al., 2016, are currently available for users to upload into, including the commonly utilised Proteomics Identification Database (PRIDE). However, these databases may often prove impenetrable to the non-expert, and variability in the type of information uploaded (e.g., peptide identity, the identity of spectral library utilised if applicable) may prove difficult to discern for even proteomics-specialised researchers. Ideally, to ensure maximum transparency, information from raw arbitrary abundance values of both constituent peptide and protein identities and, if applicable, any manipulations underpinning the quantitation performed (such as expression ratios or mean abundance per biological group) should be included at minimum if appropriate methodology is cited in-publication [63]. Encouragingly, standardised and “user-friendly” platforms, such as the increasingly popular ProteomeXChange Consortium, in conjunction with web-based tools for standardising proteomic workflows, seek to not only implement standardised guidelines for the submission of proteomic datasets but also render them accessible for non-expert researchers [63,64,65,66,67].

### 3.3. Downstream Data Analysis

In this review, we consider “downstream” analyses of proteomic data generated via the methods described above as workflows designed to extract biological meaning out of the protein expression changes reported through mass-spectrometry-based methodology (see: Figure 1: Analysis). For the researcher designing their proteomic study with the aim of elucidating how alterations in the neuronal proteome may be contextualised into neurodegenerative disease pathologies, we again strongly emphasise here the importance of resisting the urge to “cherry-pick” proteins of interest (or the candidate du jour of the field) in lieu of a more thorough unbiased analysis. Indeed, we suggest the benefit of conducting the following potential analyses by using only a relatively ambiguous identifier, such as UniPROT/SwissPROTKB accession number and accompanying abundance values, in order to effectively “blind” analyses so that any resultant protein or pathways of interest are derived from a true unbiased methodology.

As described previously, search engine-based peptide identification methods utilise a stringent match cut-off that can be adjusted per experiment. Similarly, the use of FDR throughout the identification (and quantitation) processes theoretically should reduce the number of false identifications. However, we and others suggest conducting downstream analyses with proteins identified by two or more unique peptides in order to maximise accuracy. Following this brief filtering step, the researcher may also consider whether they wish to proceed with raw abundance values or whether they should further manipulate the data; as mentioned previously, the scope of abundance variation in the proteome is difficult to visualise due to the distribution skewing close to zero. For this reason, particularly for multiplexed studies, it may be useful to work with log2FC values or to generate ratios of expression change, for example, in the disease group(s) compared to control group(s).

At this point, in the event of a two-way experiment (i.e., Group A versus Group B), it may be relevant to apply a cut-off in expression change for subsequent downstream analyses (Figure 1: “Meaningful” data cleanup). Traditionally in bioinformatics, a 50% cut-off in altered expression is a commonly accepted starting point; however, we and others argue that this threshold is reductive in the context of biological events that we demonstrate translate to phenotypic alterations in vivo [68]. Alterations in protein expression as low as 20% may have a demonstrable impact on phenotype [69,70,71,72,73]. Conversely, a knockout model may disrupt markedly impact the proteome on a global scale; in this case, a more stringent cut-off may be employed. Cut-offs applied at this, and any subsequent stage should be carefully considered in the context of the experiment profiled by the data.

For proteomic studies designed to compare more than two experimental groups, it may be beneficial to refine the dataset further in order to capture biologically relevant trends, i.e., subgroups of proteins that may address the hypothesis more directly than an analysis of the total dataset (see: Figure 1: Trend-driven analysis). Clustering-based analyses (and additional unsupervised learning approaches) have gained increasing traction in the bioinformatics field [74]; however, these are often employed toward creating a molecular atlas by grouping proteins closely related in their biological function or localisation. Instead, we demonstrate the application of the Markov Clustering algorithm based simply on the variable of expression trend between groups [75].

For example, Hesse et al. stratified a study of the post mortem Alzheimer’s Disease brain based not only on regional vulnerability (with appropriate age-matched controls) but also on different levels of the primary genetic risk factor ApoE4. Through expression profile clustering, Hesse et al. were able to identify and isolate a subgroup of proteins that tracked strongly with both ApoE4 levels and regional vulnerability in the post mortem Alzheimer’s brain from the otherwise highly complicated end-stage synaptic proteome [24]. The same application may be used to, for example, isolate trends across disease progression, through different doses of treatment or through different anatomical regions of the brain.

#### 3.3.1. Enrichment Analysis: As a Whole vs. for Individual Trends

Biological “enrichment” analyses provide a preliminary insight into the degree of overlap within an input gene list (or, in these studies, protein lists) holds against a predefined database of biological processes. These analyses are useful for obtaining information about the potential representation of particular pathways, biological processes, or cellular functions (for example) within a dataset; however, as these enrichment scores do not incorporate accompanying quantitation values (i.e., expression values), they are limited in their whether an “enriched” pathway is activated or inhibited, for example.

In this manuscript, we focus briefly on the enrichment analysis capacities available through the two most common online enrichment tools, DAVID [76,77] and STRING [78], each of which hosts its own predefined and periodically updated libraries. To perform enrichment analysis, the user uploads their list of, for example, proteins as a single list of standard identifiers such as UniPROT/SwissProtKB Accession numbers, FASTA headers, etc. It is possible to convert protein identifications into corresponding gene names within software such as DAVID; however, in conducting enrichment analysis of proteomic data, the researcher should be prudent in determining whether a conversion is entirely successful, as conversion capacity depends on the accuracy and version of the database curation. It is suggested that at this point, the researcher should check to see whether some entries may require manual conversion (for example, through searching for corresponding gene names in the UniPROT database) or whether conversion should be completed against multiple species databases, particularly in the case of multiplexed studies where peptide identification has been searched against multiple species in a search engine such as MASCOT.

DAVID and STRING provide a number of enrichment analysis options, including the most commonly utilised Gene Ontology (GO) term enrichment analysis. GO terms may be simplistically defined as pre-set gene groups classified based on their functional characteristics (e.g., “ER-Golgi trafficking”), with each unique GO term assigned also featuring a subdivision into three sub-categories of “biological processes”, “cellular components”, and “molecular functions”, Enrichment of genes (or proteins) within a dataset for GO terms is calculated from a number of statistical tests including most commonly a Fisher’s exact test [76,77] and the resulting “enrichment score” may be used to infer information about the degree of representation a specific biological process holds within the data.

In terms of application to neurodegenerative disease research, it is again important to note that enrichment analysis, while useful for gaining a preliminary insight into the biological processes potentially involved in a dataset, ultimately provides limited information on a pathway level as it does not incorporate quantitative information alongside protein lists. However, it may be employed in other uses, such as determining success in a subcellular fractionation protocol (for example, high enrichment for mitochondrial processes following isolation of synaptic mitochondria) prior to a validation experiment such as western blotting [79].

#### 3.3.2. Network and Pathway Analysis

Within the context of proteomic studies, the systems biology field has emerged on the basis of considering that the ultimate biological function of altered protein expression is not simply an alteration(s) in the known mechanism(s) of action of a singular protein(s), but rather that protein(s) themselves exist within larger biological network(s), with alterations within network components producing greater implications upon a cellular or organismal phenotype (see Figure 1: Contextualisation). Within the context of proteomics-based systems biology applied to the study of neurodegenerative disease, we briefly describe biological networks derived from protein–protein interaction studies (published as affinity purification-MS experiments) or through enzyme–substrate signalling networks (inferred from MS-based profiling of posttranslational modifications on putative substrates). Both protein–protein and enzyme–substrate interaction studies provide the foundation for the construction of biological networks incorporating protein(s) of interest with capacity for inference upon, for example, a potential contribution toward synaptic dysfunction.

These networks are comprised of functional molecules or “nodes” embedded in a framework of direct or indirect interactions or “edges”- derived from aforementioned MS-based experimentation. An example of a direct “A- > B” edge in one of these networks would be Protein A having been previously discovered to interact with protein B through affinity purification-MS, whereas an example of an indirect “A- > C” edge would be Protein A having been characterised as a transcription factor, of which transcriptional targets include Protein B which has been previously demonstrated through affinity purification-MS to catalyse the activity of Protein C. For many diseases, it is interesting to contextualise how individual protein alterations identified in a bottom-up proteomics study may impact these larger biological networks. These differentially expressed proteins could then be detectable in the wet lab, from altering expression levels of a downstream protein of interest to yielding organellular dysfunction or cellular alterations visible at an immunohistochemical level. Ultimately, a systems biology approach harbours the potential to lend greater context to how alterations in the proteome produce neurodegenerative phenotypes.

In order to generate these networks, a number of freely available network generation tools are popular amongst system biologists and molecular biologists alike, with most available as packages in Python or R or else have built-in web interfaces for user-friendly accessibility [80]. Additionally, machine learning approaches have been gaining increasing traction in the systems biology field [81,82]. Similarly, biological network databases such as KEGG [83,84] and Pathway Commons [85] are freely available online and are useful for searching. However, the researcher must consider that these resources may be limited by a lag in the curation of these interaction libraries [86]. Similarly, it may be difficult to generate networks incorporating multiple species libraries for multiplexed studies that include, for example, both human and rodent protein identifications.

User-friendly network analysis software for the non-systems biologist, such as Ingenuity Pathway Analysis (IPA) (Ingenuity Systems, Silicon Valley, CA, USA), is similarly capable of generating biological networks from proteomic data as well as enrichment-style analyses incorporating protein expression values. IPA produces pathway activation or inhibition scores, as well as mapping putative upstream regulators and the broader downstream biological effects of an input protein list. These analyses harbour an advantage in being derived from the continually updated, “hand-curated” Ingenuity Knowledge database of publications. Additionally, “all-comers” network analyses applications such as IPA have also broadened their capacities to not only generate interactions between protein (or genes/transcripts) but also include the ability to incorporate additional “nodes”, such as microRNAs and even pharmacological compounds based on, for example, high throughput screening assays.

On the other hand, in recent years, several research groups, including the pioneering Barabási laboratory, have sought to design customised workflows incorporating network-based algorithms, ranging from those extracting biological network database information such as those described above (e.g., KEGG or protein atlas information) to AI-based algorithms, with known biological information such as stereotyped cytoarchitecture or the known neuronal connectome [87,88].

As always, with these network-based approaches, the researcher must consider that extrapolation into biological pathways or predictive upstream regulator analyses must be undertaken with prudent incorporation of the scientific question at hand; for example, at this point, would it provide greater insight to analyse a dataset as a whole, or to attempt contextualisation of individual trends isolated through, for example, expression profile clustering (see: Data Analysis)?

Similarly, network analyses including quantitative expression level data may prove more informative with an expression level cut-off and/or by introducing parameters such as restricting the background information to include only specific species or cell lines, or interactions including certain classes of proteins, or experimentally observed interactions. A considered analysis incorporating these more stringent parameters may also have an advantage in circumventing traditional “roadblocks” in systems analysis surrounding the computational power required to search the enormous scope of interaction database information.

Ultimately, however, any in silico results must be corroborated with appropriate validation experiments in vitro or in vivo. Indeed, this coupling of in silico predictions with a proof-of-principle demonstration through “wet lab” techniques appears increasingly imperative in the application of “network medicine.” [89]. For the neurodegenerative researcher aiming to elucidate a mechanistic contribution toward disease-specific pathogenesis from their proteomics experiment, tracing putative candidate networks across additional proteomic studies—publicly available or collaborative—profiling progressive neurodegenerative conditions may serve as an additional useful step in highlighting translational relevance.

## 4. Comparative Proteomics May Uncover Common Regulators of Neuronal Stability within and between Distinct Models of Disease

In recent years, comparative approaches to glean information from the “known” (i.e., shared phenotypes of synaptic vulnerability, pathological aggregates, etc.) in order to shed light on the unknown (i.e., degenerative-relevant cascades) have increasingly gained traction in the field [90,91]. For example, the critical role of synaptic involvement across a broad range of conditions and the exceptional vulnerability of synapses to a wide array of pathological stimuli is now well documented. Numerous studies have highlighted the critical role that synaptic malfunction and degeneration play in preclinical and early symptomatic stages of neurodegenerative conditions, including motor neuron diseases [92,93], Alzheimer’s Disease [94], prion diseases [95], Parkinson’s Disease [96], spinocerebellar ataxia [97], the spastic paraplegias [98], Huntington’s disease [99], and lysosomal storage disorders such as the CLN1-13 forms of Batten disease [70,100,101]. In all of these conditions, synaptic pathology generally occurs in advance of pathological changes occurring in other regions of the neuron (i.e., cell body and/or axon). Conversely, targeting the mechanisms identified in differentially vulnerable neuronal compartments can rescue not only the synapse but also the remainder of the neuron and potentially the whole neuromuscular system [72]. Indeed, even if the molecular cascades are correctly tracked far enough upstream, it may be possible to subsequently identify modifiers capable of influencing disease progression in every organ system examined [72,73].

It is perhaps to be expected that a number of studies have sought to exploit this conserved phenotype of synaptic vulnerability with the aim of untangling a common, pro-degenerative molecular signature promoting this synaptic phenotype across conditions considered clinically or genetically distinct. Indeed, a comparative proteomic approach, encompassing the caveats outlined previously, is well-poised to identify some of these conserved regulators of synaptic and, therefore, general neuronal stability. Furthermore, these considerations may also be employed in comparative approaches beyond determining multi-disease overlaps, including addressing potential questions regarding the suitability of genetic models or enhancing the veracity of animal model-based findings through an additional exploration into the human post mortem proteome.

### 4.1. Comparative Approaches for Complex or Unknown Aetiologies

Alignment and comparison of proteomic studies profiling otherwise unrelated neurodegenerative conditions could increase our biological understanding of potentially conserved disease mechanisms promoting neurodegeneration but could importantly aid the identification of therapeutically targetable elements whose utility would not be restricted to a single condition. This would be particularly beneficial for the majority of progressive, degenerative conditions of ambiguous genetic origin that are currently without available therapeutic options. A comparative approach also introduces a therapeutic advantage in combining multiple drug discovery fields, including the potential for repurposing previously studied and approved compounds.

A common starting point in multi-disease comparison lies in shared phenotypic patterns of differential vulnerability in neuronal populations. For example, despite their differences in age of onset in their most common forms, two motor neuron diseases generally fall on opposite ends of the heritability spectrum—the monogenetic spinal muscular atrophy (SMA) and the majority “sporadic” and adult-onset amyotrophic lateral sclerosis (ALS), as well as the 5–10% of cases classed as familial—share highly similar progressive pathologies in the lower motor neuron. Perhaps unsurprisingly, an increasing body of evidence suggests that the two most common motor neuron diseases are linked beyond symptomatic similarities toward shared elements of molecular dysregulation converging upon common core regulatory pathways [102]. For example, overexpression of SMN in ALS mouse models and in vitro appears to be modestly neuroprotective, with an improvement of neuromuscular phenotype and an increase in lifespan, and resistance to mutant SOD1[G93A] toxicity, respectively [103,104,105]. Conversely, depletion of endogenous mouse *Smn* successfully enhances disease pathogenesis in ALS mice [106]. Additionally, the SOD1[G93A] mutation, the first identified ALS-associated gene, is capable of disrupting Smn localization in vitro and in vivo [107,108]. Finally, the ALS-associated RNA-binding protein FUS has been demonstrated to not only colocalize with SMN in vitro but also directly interact with an Smn-containing complex, while several ALS-associated mutations in FUS are capable of significantly disrupting the typical axonal distribution and function of SMN in vitro [109,110].

Recently, studies into the stereotyped patterns of differential motor neuron vulnerability have identified molecular differences between motor neuron populations and have importantly also identified cross-disease modifiers of both SMA and ALS [62,111,112]. Alignment of proteomic studies profiling both ALS and SMA models has since revealed several similarities at the individual molecular to the pathway level, including elements of dysregulation encompassing RNA processing and NF-kB pathways, as well as endoplasmic reticulum-Golgi trafficking processes [61,113,114]. It is, therefore, evident that a significant degree of molecular conservation exists between SMA and ALS and that a greater identification and characterisation of these commonly dysregulated cascades may prove therapeutically beneficial.

We additionally propose that this more comprehensive comparative approach can help address the seemingly impenetrable list of uncertainties surrounding conditions for which the relevance of transgenic animal models to more genetically ambiguous human conditions remains contentious, such as in the muscular dystrophies [115] or the neuronal ceroid lipofuscinoses [116]. For example, alignment of novel or previously published proteomic studies profiling differentially vulnerable neuronal populations or neuronal sub-compartments between different mouse models of ALS [117], Parkinson’s Disease, or Alzheimer’s Disease [118] may uncover commonly dysregulated cascades serving as key drivers of disease in each condition.

### 4.2. Incorporation of Human Proteome Analysis

A comparative alignment of proteomic studies, including the incorporation of increasingly available human proteomic datasets, may also serve to enhance the translatability of findings derived from animal models. In recent years, advances in proteomics technologies have enabled the profiling of (1) both healthy and disease-associated synaptic and (2) general neuronal proteomes, including “atlases” of expression across brain regions [27,79,90] as well as other (3) neurodegenerative and neuromuscular-disease relevant tissue such as the human neuromuscular junction, and skeletal muscle proteome [9,119]. These may potentially serve as a powerful tool in overcoming the persistent challenge of translating basic research toward clinical trial successes.

A comparative insight into potentially conserved dynamics between preclinical animal models and the human proteome—limited not only to shared protein expression alterations at the cellular or compartmental level but also commonly dysregulated post-translational modifications or broader alterations on a system or pathway level—may alleviate the typical limitations of animal model studies or interpretation of human data. However, these comparisons should be designed to maximise the possibility of identifying overlaps harbouring true biological relevance. For example, while generating or incorporating post mortem disease data into a comparative study, it is prudent to consider the presence of end-stage alterations in affected neurons. For this reason, the inclusion of animal data profiling pre- or early symptomatic alterations as well as introducing additional parameters of regional vulnerability in post mortem tissue, or examination of protein alterations following genetic manipulation in vitro, may avoid false positives in identifying molecular “commonalities” attributed to multiple instances of cell death. Encouragingly, this cross-species approach has been utilised in recent studies profiling both Parkinson’s and Alzheimer’s disease post mortem tissue [120,121,122,123].

## 5. Concluding Commentary

Recent advances in proteomics technologies, particularly quantitative mass-spectrometry-driven approaches, have ushered in a new era in the potential for delineating the molecular dysregulation underpinning currently fatal neurodegenerative diseases such as Alzheimer’s Disease, ALS, Parkinson’s Disease, and most forms of the neuronal ceroid lipofuscinoses. However, several issues regarding study design, sample collection and handling, and perhaps most critically, post- mass spectrometry analysis with appropriate validation, should be considered in order to address the current “roadblock” in identifying candidates harbouring translational relevance at the therapeutic level. Moreover, a comparative approach in combining and aligning datasets of separate proteomic studies (example in Appendix A), particularly including profiles of monogenetic conditions (i.e., aetiologically straightforward) or human post mortem samples when available, while encompassing considerations described previously (e.g., gene dose, disease staging, regional vulnerability), may enhance the discovery of viable non-gene-replacement-derived therapeutic options.

## Figures and Tables

**Figure A1:**
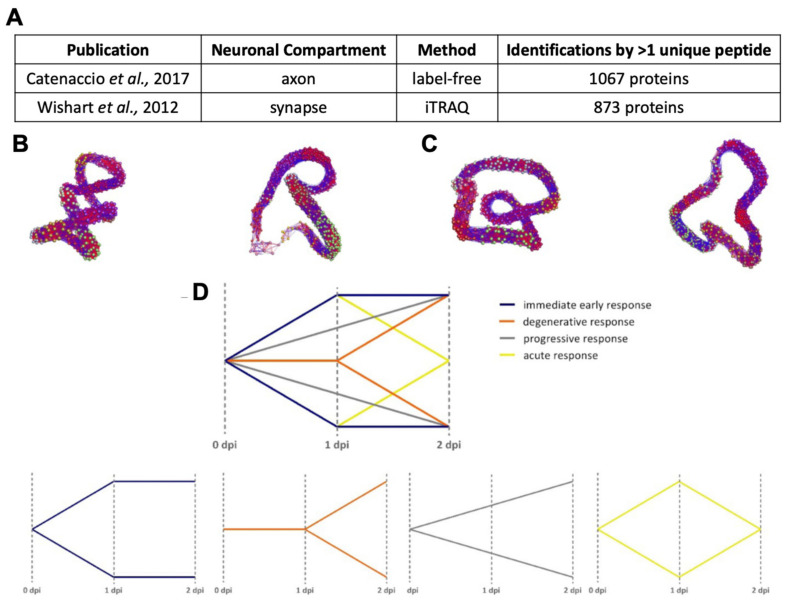
(**A**) Summary of source datasets derived from studies by [124,125] profiling, respectively, murine synaptic (following cortical lesion injury) and axonal isolates (following sciatic nerve crush injury). Table summarises labelling method employed, number of analysis-ready proteins as identified by the presence of 2 or more unique peptides, and main findings following downstream analysis as described by the authors. (**B**,**C**) BioLayout3D representation of (**B**) WT synaptic and (**C**) WT axonal protein expression status through 1 dpi and 2 dpi relative to day of injury (0 dpi). Each node represents an individual protein, and lines represent the magnitude of expression correlation between proteins across the three time points profiled in each study (0, 1, and 2 dpi). Input was clustered based on similarity in expression profile over time, and individual nodes are colourised to represent identity within output clusters. Correlation threshold is defined by Pearson r = 0.97. (**D**) Representative expression profile graphs illustrating potential responses of protein subsets following injury, indicating putative immediate early, degenerative, progressive, and acute response profiles.

**Figure A2:**
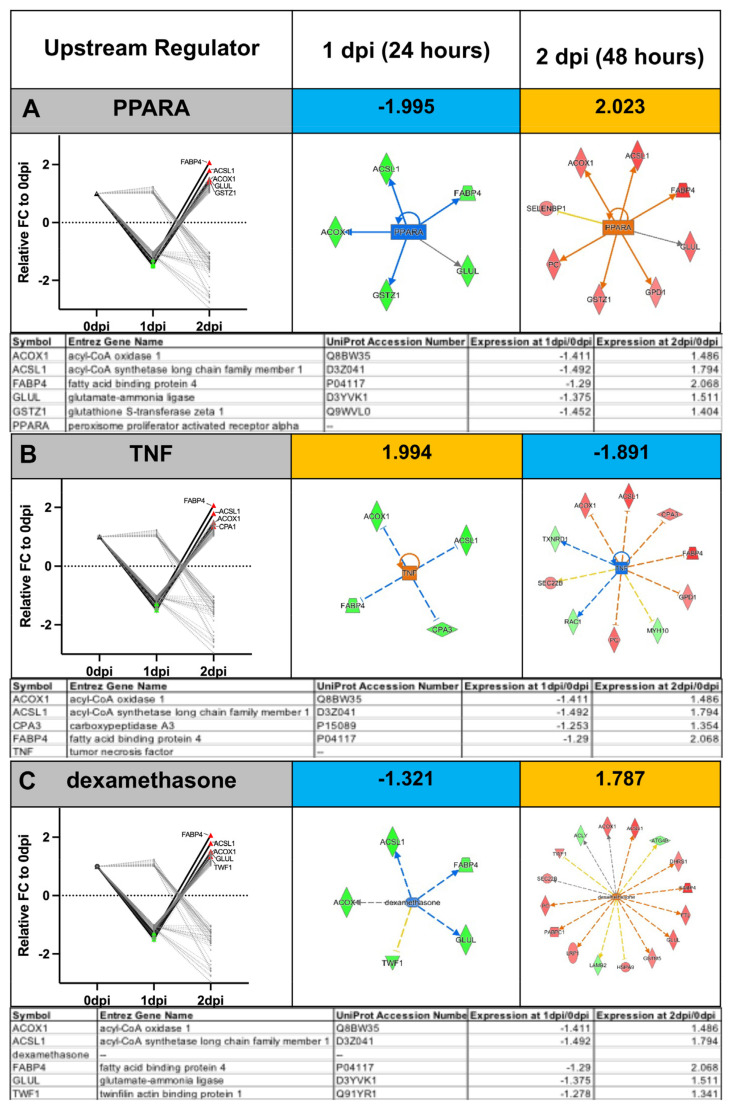
Trend-wise and compartmental dissection of expression trends highlight molecular dynamics within the axon through the injury-induced degeneration process. Top upstream regulators, (**A**) peroxisome proliferator-activated receptor alpha (PPARA) (**B**) tumour necrosis factor (TNF), and (**C**) dexamethasone exhibit opposing activation status (as indicated by +/− z-score), mediating opposing directionality of target molecules between 1 dpi and 2 dpi following axotomy. Tables and graphs highlight relative expression of regulatory candidates through 1 dpi and 2dpi compared to 0 dpi as detected by proteomics study published in [124].

**Figure A3:**
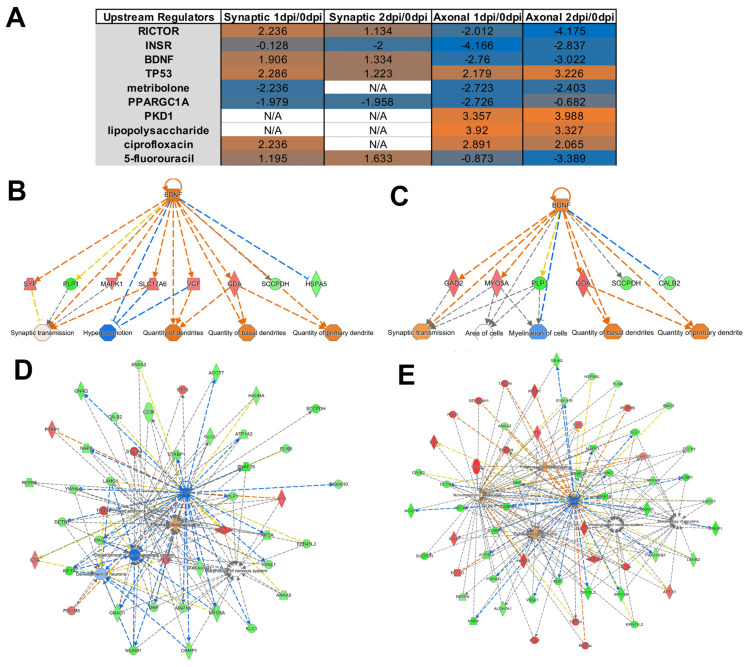
Overlay of expression profile trends within axonal versus synaptic compartments post-injury reveals predictive convergence upon known regulators of Wallerian degeneration. (**A**) Summary table of top 10 upstream regulators mediating identified biologically relevant axonal and synaptic trends as ranked by absolute z-score. Note presence of known neuronal and non-neuronal (i.e., myelinating) regulators, including RICTOR, insulin receptor, BDNF, and PKD1. (**B**) Example extrapolation of mechanistic network mediated by predicted upstream activation or inhibition of BDNF signalling in the synapse (**B**,**C**) or axon (**D**,**E**), respectively, promoting alterations in downstream protein expression as detected by respective proteomic study. As proof-of-principle, the top 5 biological functions per network consequential to expression alterations in constituent proteins represent known phenotypic outcomes. See Figure A4 for an expression summary of constituent proteins in BDNF-regulated mechanistic networks depicted in (**B**–**E**) in synapse and axon over 1–2 dpi.

**Figure A4:**
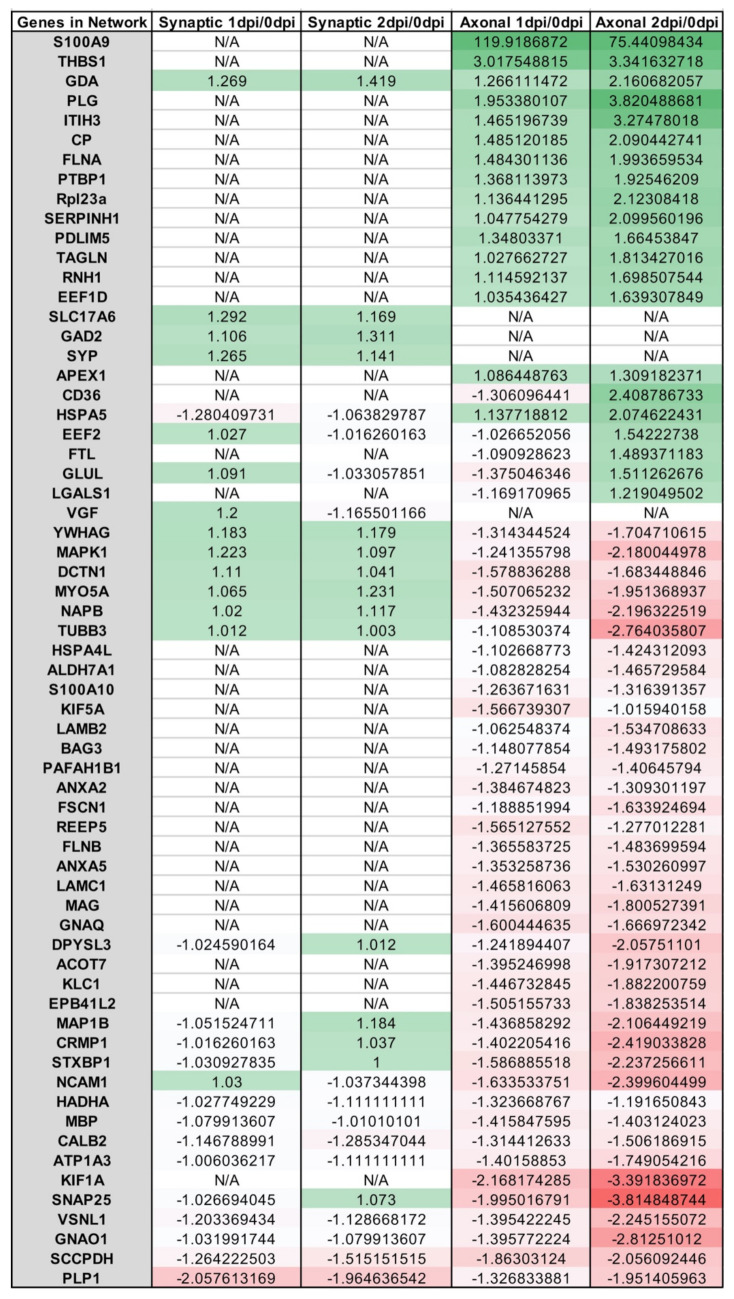
Summarised expression values of constituent proteins in BDNF-regulated mechanistic network depicted in Figure A3(B–E) in synapse and axon over 1–2 dpi.

## Data Availability

Not applicable.

## Appendix A

Here, we present an example of a “proof-of-principle” alignment of experimental data, in which the original datasets have been processed in a standardised manner encompassing the considerations previously described in this manuscript. Source datasets may be found in [124,125]. Briefly, the comparison for this study was designed to determine whether it was possible to identify conserved regulators of neuronal stability across distinct neuronal compartments by identifying pro- or anti-degenerative molecular alterations occurring in both the synaptic and axonal proteome. We suggest that incorporating aspects of the methodologies presented below may be employed to enhance the analysis of datasets profiling disease or injury-induced degeneration.

### Appendix A.1. Methods

#### Appendix A.1.1. Source Datasets

Neurons are organised in specialised subcomponents, including axons and synapses. Regarding the degeneration process, these parts behave independently from each other, and synapses appear to be specifically vulnerable to triggering events [126]. Key observations highlighting segregation of molecular mechanisms regulating axonal and synaptic degeneration come from studies of the natural murine mutation slow Wallerian degeneration mutation (WldS), comprising a fusion of the endogenous mouse genes Ube4b and Nmnat1. Overexpression of the slow Wallerian degeneration protein (WldS) in homozygous mice allows the structural and functional delay of axonal degeneration, age independently, for 10 days or more, compared to degeneration and clearance within 48 h of injury in adult wild-type mice [69,127].

Profiling the proteome of the distal compartments (axon and synapse) of WldS mice versus wild-type mice over a time course of wild-type degeneration (0, 1, and 2 days post-injury (dpi)) following injury may therefore uncover regulators of neuronal stability in injury-induced degeneration. The following datasets were therefore used for comparison (Figure A1): synaptic dataset of Wld(s) mouse versus wild-type mouse following axotomy; experiments described in [125] with the datasets for axon degeneration [124] in order to investigate potential regulatory candidates after induced injury. The first dataset investigated synaptic degeneration induced by a cortical lesion model, and the proteins were iTRAQ-labelled for mass spectrometry analyses [125]. The second dataset utilised a sciatic nerve injury model to induce axonal degeneration, and the proteins were identified and quantitated using label-free proteomics [124]. Both datasets contain the relative ratios for WT and Wlds mice over three time points at specific Days Post Injury (0 dpi, 1 dpi, 2 dpi).

#### Appendix A.1.2. Standardisation for Alignment

Raw data sets containing all identifications for either synaptic or axonal degeneration were processed in the same manner. Firstly, files were filtered in Microsoft Excel (Windows 2010) to produce columns containing the following identifying information: accession number, gene name, and description. Thereafter, the data values for each time point were converted into relative ratios of expression against the first time point before injury (i.e., WT0/WT0, WT1/WT0, WT2/WT0, Wlds0/Wlds0, Wlds1/Wlds0, Wlds2/Wlds0). These files containing time point ratios and identification columns were subsequently used for alignment and pathway analysis.

#### Appendix A.1.3. BioLayout Express3D

The BioLayout Express3D software [75] was used for the visualization, integration, and analysis of the network graphs derived from each dataset (synapse and axon degeneration) in order to define the relationship between protein clusters [75]. Individual Excel files were uploaded into BioLayout Express3D, and the relative expression profiles from time point 0 to 2 dpi were analysed. By means of the Markov clustering algorithm (MCL) within BioLayout Express3D, the graphs were divided into distinct clusters possessing similar expression profiles, and the individual proteins were represented over time (Figure A1). The generated visual networks were subsequently utilised to identify response patterns correlating with distinct steps of the degenerative process known time course of structural degeneration in wild-type mice, such as that described in [124]. For this, proteins with a similar expression profile over time, regardless of the direction of expression, were assigned to the same response pattern (see the summary of 4 profiles in Figure A1). Proteins constituting the “degenerative” cluster in both synapse and axonal datasets, i.e., those that exhibited a steady increase or decrease in expression from 0 to 1 to 2 dpi (see Figure A1D for a visual demonstration of protein trends), were then subjected to pathway analysis (see Appendix A.1.5. and Figure A2 and Figure A3).

#### Appendix A.1.4. DAVID

The *Database for Annotation, Visualization, and Integrated Discovery* (DAVID) is a freely accessible bioinformatics tool used to determine enrichment of functional annotations ascribed to groups of genes, transcripts or proteins. In this analysis, proteins of a corresponding cluster identified through unbiased expression profile clustering performed through BioLayout Express3D (see Appendix A.1.3) were submitted as genes into DAVID Bioinformatics Resources 6.8 and converted into a DAVID ID list against *Mus musculus*. Analyses were performed for the total gene list and the 4 BioLayout Express3D clusters (1) immediate early response, (2) degenerative response, (3) progressive response, and (4) acute response (summarised in Figure A1), were determined for both synaptic and axonal degeneration data. The individual lists were further analysed by DAVID Bioinformatics in terms of functional annotations that are ranked as a DAVID enrichment score. A significant difference is reached with an enrichment score of >1.3 (which is on equal terms with *p* < 0.05) [76].

#### Appendix A.1.5. Ingenuity Pathway Analysis

The Ingenuity Pathway Analysis software applies a “hand-curated” database to investigate complex proteomics data, including cellular and molecular pathways involved in statistically significant changed protein expression after injury. The application generates networks of interacting proteins predicting downstream effects and helping to identify potential regulator candidates based on experimentally reported interactions derived from published publications. Using IPA, the relative ratios of expression changes were uploaded in terms of a gene list. Then the standard settings were set to ‘direct and indirect interactions’ and ‘experimentally observed data only’. For networks generated by IPA, the number was limited to 35 member molecules and a maximum of 10 networks per analysis. The threshold was set to 20% for the axon and 15% for the synapse data set. This difference in threshold was set to account for the difference in methodological sensitivity, sample type origin, coverage, and differentially detected identifications.

Predicted activation z-scores of putative upstream regulators, as presented in Figure A2 and Figure A3, were calculated by weighing the predicted expression change of target molecules within the regulator’s interactome as defined by the Ingenuity Knowledge Database against the actual expression change of target molecules reported in input datasets. The top absolute z-scores per analysis, as described in the following text, are presented in Figure A2 and Figure A3. For Figure A2, these calculations were performed on proteins identified in wild-type mouse axonal compartments [124] to exhibit a “degenerative” expression profile (Figure A1D) from 0 to 2 dpi; activation z-scores at 1 and 2 dpi, as well as the expression ratio of each protein within axonal “degenerative” cluster constituting the regulator prediction, are also presented. For Figure A3, these calculations were performed on proteins identified in *both* wild-type mouse axonal [124] and synaptic [125] datasets to exhibit a “degenerative” expression profile (Figure A1D)) from 0 to 2 dpi. An activation z-score > 2 or <−2 is considered statistically significant. *p*-values of overlap derived from a Fisher’s exact test were derived from a Fisher’s exact test calculating overlap between molecules in each respective input dataset and the number of molecules comprising the known interactome of each regulator as defined by the Ingenuity Systems Database.

In graphical format for networks displayed in Figure A2 and Figure A3, target molecules present within each proteomic dataset predicted to be activated or inhibited by reported regulators were visualised in relation to their associated predicted regulator and were colourised with intensity of colour corresponding to magnitude of change; red represents upregulation between 0 to 1 to 2 dpi within each respective model, while green represents downregulation. Solid connecting lines represent a direct interaction, while dashed connecting lines indicate an indirect interaction.

### Appendix A.2. Example Results

Clustering protein expression over 1–2 dpi according to known ultrastructural changes in the wild-type neuron reported in [124,125] enables the isolation of “degenerative,” “progressive,” “immediate early”, and “acute” protein expression profiles based on accompanying observations of axonal and synaptic at the morphological and functional level following injury [124]. For example, axotomy-induced degeneration in the wild-type sciatic nerve, previously used as a molecular baseline for which to compare correlates of resistance to degeneration in the WldS mouse model, induces early morphological alterations including Schwann-cell-dependent fragmentation detectable within 24 h of injury (1 dpi), whereas subsequent disintegration of fragments. Thus, early alterations occur within the first 48 h of injury. The following examples in stratifying data by their correlation with various steps in the cytoarchitectural breakdown of the distal compartments of the neuron may be considered in designing analyses of multiple tissues or time points (Figure A1).

As an example, it is possible to map “degenerative” alterations (i.e., small alterations from 0 dpi to 1 dpi, then increasing or decreasing in expression >20% from 1 dpi to 2 dpi; refer to Figure A1D) through opposing directionality in predicted upstream regulator activity over 1–2 dpi (Figure A2). It is then possible to perform a comparative overlay of degenerative profiles in both synapse and axon through 1–2 dpi (Figure A3). For example, we demonstrate here that these distinct studies profiling the wild-type axon and synapse following injury-induced degeneration report conserved protein alterations upstream convergence upon known signalling mediators of injury-induced degeneration (Figure A3A). While these results have been compiled as an example, this comparative approach may be employed by the researcher to identify novel upstream regulators within a degenerative experiment or else to map known upstream regulators to putative novel downstream effector molecules, as in Figure A3F.
